# The impacts of sport emissions on climate: Measurement, mitigation, and making a difference

**DOI:** 10.1111/nyas.14925

**Published:** 2022-11-15

**Authors:** Robert L. Wilby, Madeleine Orr, Duncan Depledge, Richard Giulianotti, George Havenith, Jamie A. Kenyon, Tom K. R. Matthews, Stephen A. Mears, Donal J. Mullan, Lee Taylor

**Affiliations:** ^1^ Department of Geography and Environment Loughborough University Loughborough UK; ^2^ Institute of Sport Business Loughborough University London London UK; ^3^ International Relations, Politics and History Loughborough University Loughborough UK; ^4^ School of Sport, Exercise and Health Sciences Loughborough University Loughborough UK; ^5^ Environmental Ergonomics Research Centre Loughborough University Loughborough UK; ^6^ Department of Geography King's College London London UK; ^7^ Department of Geography Queen's University Belfast Belfast UK

**Keywords:** carbon footprint, emissions, meta‐analysis, mitigation, sport

## Abstract

As a global industry, sport makes potentially significant contributions to climate change through both carbon emissions and influence over sustainability practices. Yet, evidence regarding impacts is uneven and spread across many disciplines. This paper investigates the impacts of sport emissions on climate and identifies knowledge gaps. We undertook a systematic and iterative meta‐analysis of relevant literature (1992–2022) on organized and individual sports. Using a defined search protocol, 116 sources were identified that map to four sport‐related themes: (1) carbon emissions and their measurement; (2) emissions control and decarbonization; (3) carbon sinks and offsets; and (4) behavior change. We find that mega sport events, elite sport, soccer, skiing, and golf have received most attention, whereas grass‐roots and women's sport, activity in Africa and South America, cricket, tennis, and volleyball are understudied. Other knowledge gaps include carbon accounting tools and indicators for smaller sports clubs and active participants; cobenefits and tradeoffs between mitigation‐adaptation efforts in sport, such as around logistics, venues, sports equipment, and facilities; geopolitical influence; and scope for climate change litigation against hosts and/or sponsors of carbon‐intensive events. Among these, researchers should target cobenefits given their scope to deliver wins for both climate mitigation and risk management of sport.

## Graphical abstract

Sport makes potentially significant contributions to climate change through both carbon emissions and influence over sustainability practices. Evidence regarding impacts is uneven and spread across many disciplines. This systematic review and meta‐analysis investigates the impacts of sport emissions on climate and identifies knowledge gaps. Researchers should target co‐benefits given their scope to deliver wins for both climate mitigation and risk management of sport.

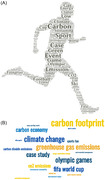

## INTRODUCTION

The global sports market was worth over US$500 billion in 2020 and is forecasted to exceed US$700 billion by 2026.^1^ Revenues are generated from a range of activities^2^ spanning sports retail (36%), infrastructure, consumables, and gambling (26%), professional sport ticketing, sponsorship, TV rights, and player transfers (26%), clubs and gym membership fees (15%). Individual tournaments can generate both immense costs and proceeds for hosts. For instance, the Tokyo 2020 Olympic Games cost US$35 billion^3^ and generated about US$5 billion in revenue, whereas the Qatar 2022 FIFA World Cup could cost $220 billion^4^ and is expected to return US$17 billion to the host economy.^5^ The cost is comparable to the entire 2021/22 budget ($230 billion) of the Department of Health and Social Care in England.^6^

Popular sports attract massive international audiences and have large numbers of participants.^7^ For example, FIFA estimates that soccer has 265 million active players worldwide and a fan base of 4 billion people—half the global population. Other sports with huge followings include cricket (2.5 billion), ice and field hockey (2 billion), and tennis (1 billion). Given the socioeconomic and cultural significance of sport, there has been growing attention to the environmental impact of the sector,^8^, ^9^ as well as to the possibilities for raising awareness and promoting greater sustainability.^10^ Recurrent themes within the literature include management practices, fan engagement and behaviors, facilities management, marketing and communication, performance monitoring and evaluation, and corporate social responsibility.^11^ These involve diverse disciplines, such as architecture, geography and environmental studies, sport management, tourism, urban studies, and others.

Increasingly, it is recognized that there is a bidirectional relationship between sport and climate because sport both affects *and* is affected by the climate system.^12^ These associations are shaping a new subdiscipline of “Sport Ecology” that embraces *human interaction with the natural environment, broadly and within a sport management context* (Ref. 13, p. 510). Hence, recent lines of inquiry are assessing the impacts of climate change and extreme weather on the sports industry,^14^, ^15^ or the adaptation of facilities, athletes, and fans to new climate conditions.^16^, ^17^ This research examines how climate change affects the viability and fairness of sports,^18^, ^19^, ^20^ as well as the suitability of cities to host future winter and summer Olympic and Paralympic Games.^21^ Others focus on the links between climate conditions and the performances of professional and amateur athletes.^22^, ^23^ Weather conditions can also affect the inclination to exercise and adversely impact participant health.^24^, ^25^ For extreme sports, such as mountaineering, severe weather can make the difference between extraordinary triumph,^26^ life, or death.^27^

These examples illustrate some of the varied ways in which climate change impacts sport. A recent structured review of literature on the subject grouped research into five major themes: (1) heat impacts on athlete and spectator health; (2) heat impacts on athlete performance; (3) adaptive measures taken in sport; (4) suitability of various cities for event hosting; and (5) benchmarking conditions for sport and defining safe playing conditions for competition.^28^ No such review exists for the impacts of sport on climate, apart from a preliminary estimate, suggesting that annual global emissions from the sport could be of the order of 350 million tCO_2_e.^29^ This equates to about 1% of all emissions linked to energy and cement production^30^ in 2019.

Aside from the initial estimate—and a review of associations between physical activity and climate, including emissions^12^—there is no synthesis of literature devoted exclusively to the impact of sport on climate. Although global emissions by sport are modest compared with other sectors, such as energy, transport, and manufacturing, they are still worthy of attention because mega sport events (MSEs), organized leagues, and mass participation sports can be “hotspots” of direct and indirect greenhouse gas emissions. Moreover, the UN Framework Convention on Climate Change (UNFCCC) *Sports for Climate Action* ^31^ recognizes that sport has an important role in achieving global climate change goals through peer‐to‐peer learning, sharing good practice, and development of new tools and collaboration. However, the strategy document^32^ is silent about the underpinning evidence needed to support these initiatives. Global emissions by sport are also uncertain because such data are typically aggregated by country or sectors that do not specify sport.

Here, for the first time, we undertake a systematic, meta‐analysis of peer‐reviewed and gray literature on the impacts of sport emissions on climate. Following Orr et al.,^28^ we apply search criteria to elucidate coherent themes and knowledge gaps around organized sport. However, we expand their scope by including research about the 25 most popular sports in the world (according to spectator numbers and/or levels of participation). The following section describes the protocols used to structure our narrative literature review^33^ of the sport–climate nexus. Qualifying material is then consolidated into emergent themes which are discussed in turn. This helps to identify knowledge gaps and opportunities for the sports industry to better engage with the climate agenda as envisaged by the UNFCCC.

## METHODOLOGY

We implemented a five‐step, iterative procedure for sifting literature with a view to drawing out key themes and evidence.^28^, ^32^ The steps were as follows:

1. Define the research questions (RQs).
2. Specify the inclusion criteria for literature.
3. Develop the review protocol and search terms.
4. Remove duplication and check eligibility.
5. Codify screened articles by sport, theme, location, methodology, and source.

For step 1, two RQs were defined:

RQ1: What evidence is available on the emissions‐related impacts of organized and major participatory sports on climate?
RQ2: What evidence is available on the actions being taken by organized and major participatory sports to reduce their emissions‐related impacts on climate?

In step 2, we searched for literature that spans the breadth of sporting entities and events (i.e., tournaments, leagues, organizations, teams, venues, participants, and spectators). In each case, the article title or abstract had to make a direct reference to the impact of sport on climate, thereby excluding papers addressing broader sustainability concerns, such as around resource consumption or environmental damage. However, the latter were retained if there was an explicit mention of an associated carbon footprint (CF). Articles were also included if they provided empirical evidence, carbon accounting tools and methods, or conceptual analysis based on empirical data gathered by others. Only articles about emissions of directly and indirectly radiatively active gases^34^ were considered (i.e., direct: carbon dioxide [CO_2_], methane [CH_4_], nitrous oxide [N_2_O], hydrofluorocarbons [HFCs], perfluorocarbons [PFCs], sulfur hexafluoride [SF_6_], and nitrogen trifluoride [NF_3_]; indirect: nitrogen oxides [NO_x_], carbon monoxide [CO], volatile organic compounds [VOCs], and sulfur dioxide [SO_2_]).

Step 3 of our protocol specifies the databases, search period, and terms. Two databases were examined for articles, book chapters, conference proceedings, theses, and reports meeting the step 2 criteria, namely: the Web of Science and Google Scholar (GS). The latter enables the capture of gray literature that might otherwise be overlooked by the other database. Our 30‐year search period (January 1992–July 2022) effectively spans the literature on the impact of the 1992 Barcelona Olympic Games through to preparations for the 2022 Qatar FIFA World Cup. However, no relevant literature was found prior to the year 1998. GS searches were viewed as far as the first 500 entries, ordered by relevance. The Sport Ecology Group archive^35^ and reference lists of key sources were also swept for overlooked materials.

Three sets of search terms were applied to article titles (see Supporting Information). Set (A) covers literature associated with greenhouse gas emissions (including aerosols) and mitigation activities. Terms such as “Nationally Determined Contribution*,” “NDC*,” “tier*,” and “scope*” were used in preliminary searches but eventually omitted from the final set because no records were found. Set (B) replicates the terms used by Orr et al.^28^ to capture literature on organized, competitive sports. Set (C) focuses the search on the 25 most popular sports worldwide—either by the number of spectators or active participants.^36^ “Running,” “Marathon,” and “Parkrun” were added in view of mass participation in these activities globally,^7^ but “Walking” and “Hiking” were regarded as recreational activities rather than competitive sports, so were excluded. Later, we will return to this issue of terminology around what is defined as recreation and what is a sport, noting that our Set (C) includes “Ski*” given the deep contradictions about climate between participant attitudes and behaviors.

In step 4, duplicate articles were excluded based on their titles or where a conference proceeding replicated a journal output. Ineligible materials were rapidly filtered when this was obvious from the title or abstract. For example, an article title “*Does signaling* ***mitigate*** *the cost of agonistic interactions? A test in a* ***cricket*** *that has lost its song*”^37^ meets the search criteria and contains two of the key terms but is clearly unrelated to the impact of sport on climate. An article on “Optimal break structures and cooling strategies to **mitigate** heat stress during a **Rugby League** match simulation”^38^ uses the term mitigate, yet the abstract confirms that this article is about adapting to heat rather than mitigating impacts on climate, so does not address our RQs and was omitted. Studies of air quality were only included if there is a cobenefit from carbon emission controls (the primary motivation) rather than when driven by human health concerns (e.g., Hu et al.^39^).

Finally, step 5 codifies short‐listed articles using key terms and phrases to identify themes for subsequent narration. This was a quasi‐objective and iterative procedure based on expert judgment and informed by word cloud analysis. The aim was to reduce the final set of sources into a representative but manageable number of topics rather than groupings by sport, tournament, or organization. Inevitably, there is overlap between themes, but code counts provide an indication of overall research emphasis, as well as some areas for development. Articles were also subclassified by sport, location, methodology of analysis, and source. Note that we did not attempt to cluster papers by scope (i.e., direct or owned emissions [Scope 1]; indirect emissions from purchased energy [Scope 2]; or indirect emissions from all other activities [Scope 3]) because such tiers apply only to a subset of articles.

### Emergent themes

#### Overview

The database searches (based on step 3) yielded 309 articles from Set (A) with (B), and 118 articles from Set (A) with (C). After removing duplicates and screening for relevance to our RQs (step 4), the final set of materials was reduced to *n* = 116. Given the relative infancy of sport‐climate research, this sample size is commensurate with other meta‐analyses of emissions from, for instance, freight transport^40^ (*n* = 81) or the tourism industry^41^ (*n* = 398).

**FIGURE 1:**
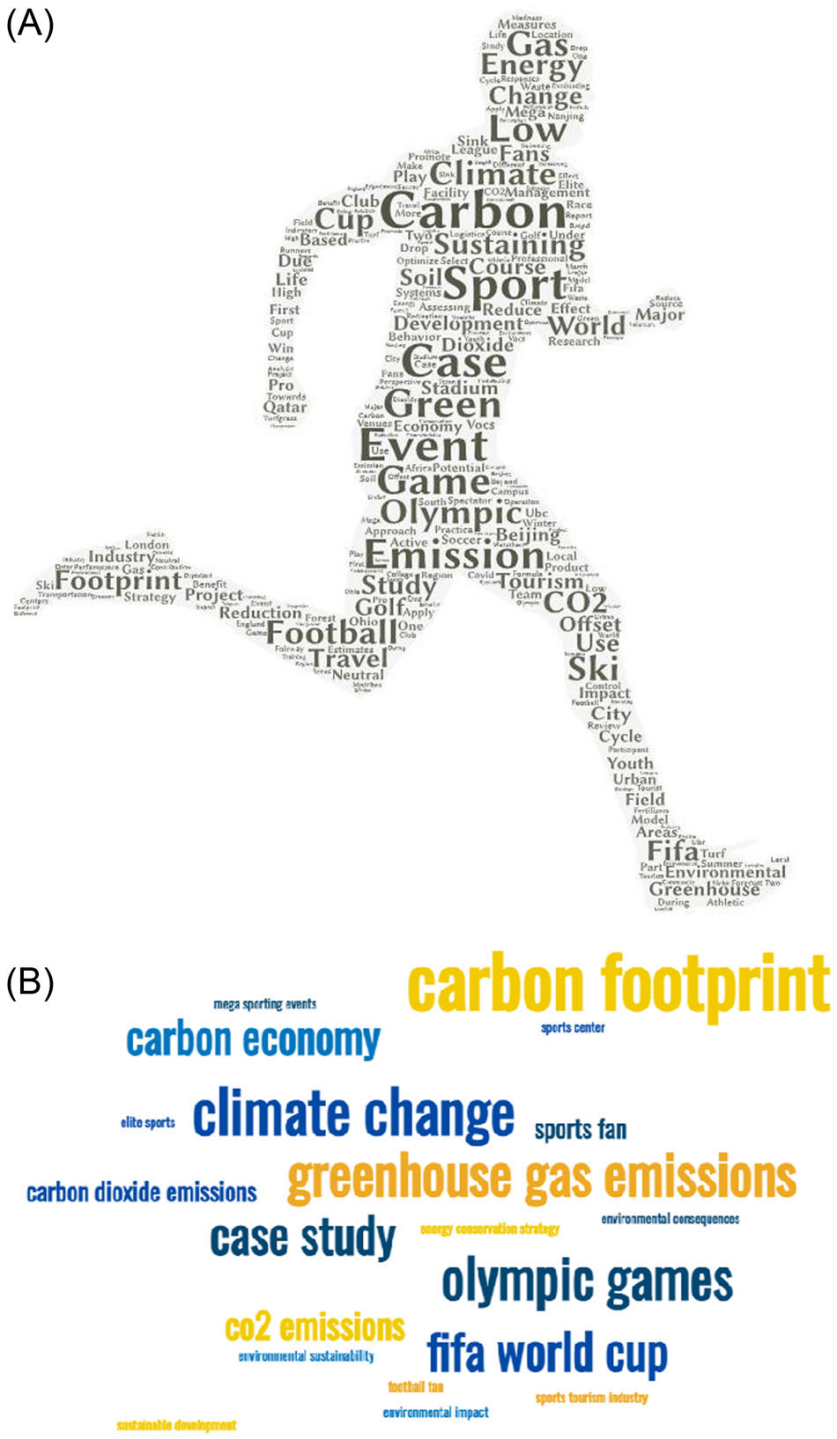
Word clouds drawn from titles (*n* = 116) for (A) most frequently used words (occurring at least twice) by WordArt [https://wordart.com/] or (B) 20 most commonly used words or phrases by Monkey Learn [https://monkeylearn.com/word‐cloud/].

We find that the six most frequently occurring words were “carbon” (*n* = 50), “sport” (*n* = 42), “emission” (*n* = 26), “sustaining” (*n* = 23), “Olympic” (*n* = 19), and “climate” (*n* = 16) (Figure 1A). Excluding place names, some of the most frequently returned phrases (comprised of two words or more) were “carbon footprint” (*n* = 12), “golf course” (*n* = 7), “Olympic Games” (*n* = 7), and “FIFA World Cup” (*n* = 6) (Figure 1B). The majority of the material was published in peer‐reviewed journals (*n* = 86), conference proceedings (*n* = 11), or reports (*n* = 7). The most favored academic journals—with four articles in each—were the *Journal of Cleaner Production*, *Journal of Sport and Tourism*, *Sport Management Review*, and *Sustainability*.

**FIGURE 2:**
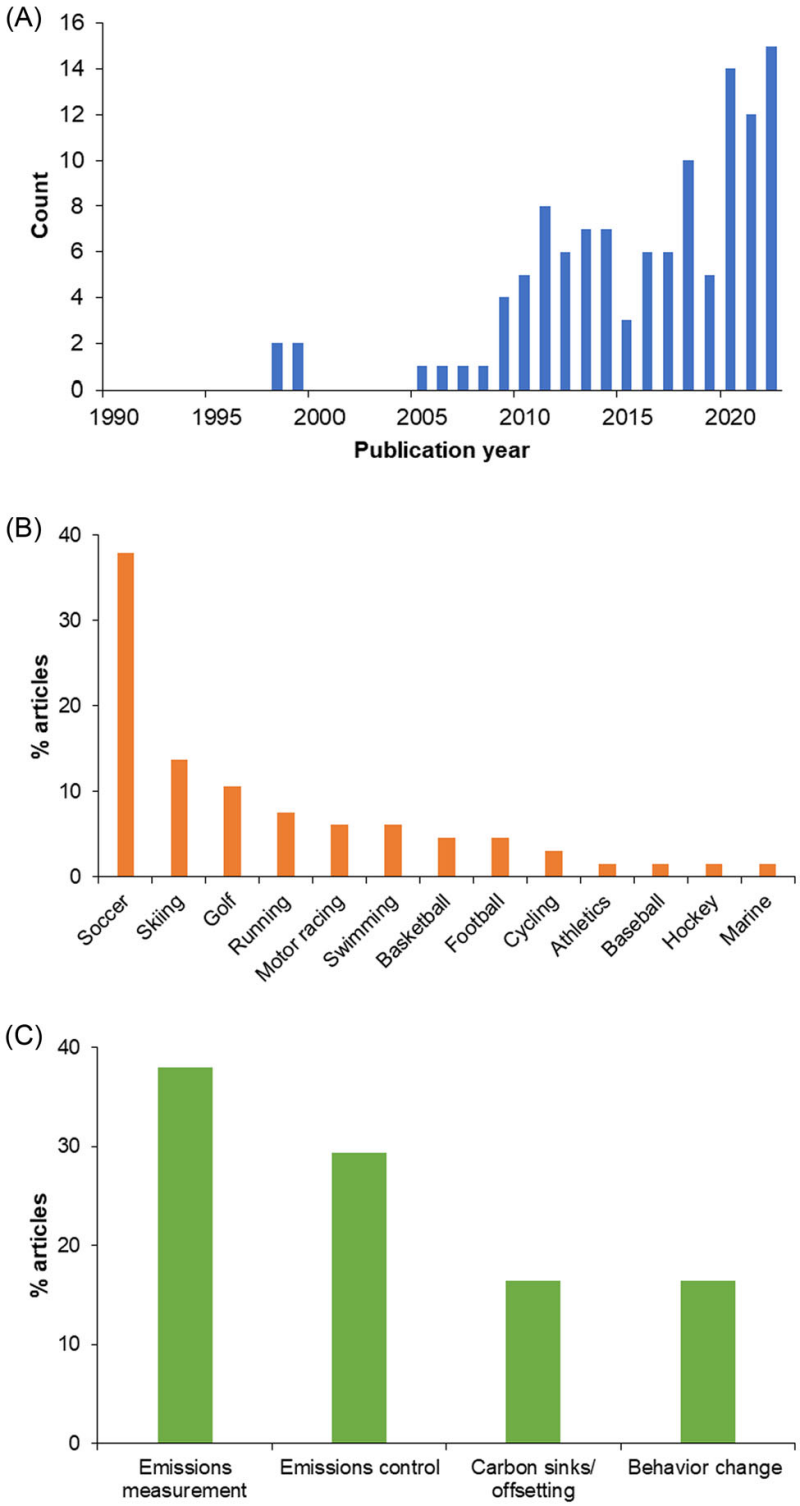
Coverage of identified literature by (A) year of publication (*n* = 116), (B) sport (*n* = 66), and (C) major theme (*n* = 116). Football is for American and Australian rules.

There are very few publications about the impact of sport on climate prior to 2009 (Figure 2A). This year was notorious for the Climategate hack^42^ and subsequent Copenhagen Summit which failed to meet earlier expectations of a legally binding, multilateral climate treaty. Beijing 2008 was the first Games to be planned after the International Olympic Committee (IOC) adopted the “environment” as its third pillar in 2001. Although Athens 2004 and Torino 2006 took some early steps, Beijing was the first to really engage with the sustainability agenda, including by partnering with Greenpeace. Hence, there are good reasons why academics began tracking the sport–climate nexus after Beijing. The volume of research output is rising, but annual numbers of sources remain modest and focused on a few sports and regions. Four sports account for 70% of the articles where the event is identifiable, namely, soccer, skiing, golf, and running (Figure 2B). Motor racing and swimming each have four sources, with American football and basketball at three articles apiece. Some globally popular sports, such as cricket and tennis, are conspicuously absent.

The study sites are mainly in Europe (25%), North America (22%), or Asia (20%), with very little representation of Africa (2%), South America (2%), or Australasia (3%)—broadly reflecting the geographic distribution of those researching Sport Ecology. The preferred methods of analysis are modeling (34%), empirical/experimental/laboratory‐based (31%), conceptual/review/opinion (29%), or survey/qualitative (6%). Numerous articles on air quality were found, but the majority (*n* = 37) were omitted from the final list because they were not principally about protecting the climate per se. Four topic clusters were then discerned among the remaining materials, listed in order of frequency: carbon emissions measurement (38%); emissions control/decarbonization (29%); carbon sinks/offsetting (16%); and behavior change (16%) (Figure 2C). These themes are elaborated in turn below.

### Carbon footprints

A CF may be defined as the total radiatively active greenhouse gas emissions (as CO_2_ equivalent) that can be directly (Scopes 1 and 2) or indirectly (Scope 3) attributed to an organization, activity, product, or asset life‐cycle. Emissions may be apportioned in various ways, but this is an essential first step for benchmarking and then reducing CFs.^43^ The methodology used by the IOC views greenhouse gas accounting as a four‐stage process of: (1) measuring emissions of the “Games project” (organization, services, and products); (2) understanding which activities contribute most to emissions; (3) taking action to reduce emissions in the most cost‐effective way; then (4) inspiring others and raising awareness about sustainability.^44^

Table 1 provides some indicative CFs for selected units of assessment, all converted to a common currency of metric tons of CO_2_e to facilitate comparison. Per capita emissions are given for selected countries to give a relative sense of the carbon cost of the various activities. Even so, evaluations are not straightforward because of the various carbon accounting methods and boundary conditions used. The extraordinary CFs of MSEs may capture attention, but even a single match, such as the 2003/04 FA Cup Final (soccer) in Cardiff, had a CF equivalent to the annual emissions of ∼110 (UK) citizens.^45^ Sports equipment, such as running shoes, may appear to have a modest impact until it is realized that ∼1 billion pairs are sold worldwide annually (although it is uncertain how many of these are actually used for running).

**TABLE 1:** Example of CFs for selected sports, equipment, facilities, and events expressed as tons CO_2_e

Scope	Unit	Tons CO_2_e	Source
Emissions from participant training, competitions, day trips, and vacations (per capita per year)	Climbing	1.156	Wicker^46^
	Diving	2.841	
	Field hockey	0.874	
	Fitness (gym)	0.228	
	Golf	2.195	
	Soccer	0.337	
	Tennis	0.243	
	Triathlon	0.775	
Emissions from equipment and facilities (life‐cycle)	Pair of skis	0.045	Luthe et al.^146^
	Polyester sports T‐shirt	0.082	Wu^49^
	Running shoes (pair)	0.014	Cheah et al.^147^
Emissions from facilities use and maintenance (various units)	Golf green (ha^–1^ year^–1^)	6.2‒7.4	Tidåker et al.^53^
	Golf fairway (ha^–1^ year^–1^)	0.8‒4.9	
	Golf rough (ha^–1^ year^–1^)	−0.6 to 1.4	
	Sports field^a^ (ha^–1^ year^–1^)	13.1	Riches et al.^52^
	Stadium (per capita event)	0.015	Hedayati et al.^50^
Emissions for sporting tournaments and events (per event)	2003/04 FA Cup Final	560	Collins et al.^45^
	2019 Formula 1 season	256 × 10^3^	Formula 1^148^
	London 2012 Games	3.3 × 10^6^	Chestney^149^
	Rio 2016 Games	3.6 × 10^6^	Rio2016.com^150^
	Tour de France Stage	170.3‒193.3	Collins et al.^151^
	US college football season	38.7 × 10^3^	Cooper^58^
Emissions per capita in 2019 (per annum)	Australia	15.2	World Bank^152^
	Brazil	2.1	
	China	7.6	
	Nigeria	0.6	
	UK	5.2	
	US	14.7	

Abbreviation: CF, carbon footprint.

^a^ Unfertilized plot monitored for 31 days.

As evidenced by Table 1, CFs can be calculated for people (e.g., individual participants,^46^ teams,^47^ and fans^48^); equipment used to play sport (e.g., rackets, boats, skis, nets, bats, balls, footwear, and clothing^49^); facilities construction and operation (e.g., stadiums,^50^ athletics tracks,^51^ cricket and football playing fields,^52^ golf fairways,^53^ and athletes’ villages^54^); and sporting events (e.g., individual races,^55^ league matches, FIFA World Cups, and Olympics). Others have derived CFs for selected sports (e.g., travel for skiing^56^ and Marathon competitors^57^) or for associated spectator activities (involving hotels, transport, consumables, and waste management^58^). For instance, a study of 20 sports in Germany showed that nature‐based, individual sports (such as surfing) have larger CFs than other individual or team sports when assessed on emissions linked to training, competitions, day trips, and vacations.^43^

Data on emissions may be gathered via plot and field experiments,^59^ remote sensing,^60^ questionnaires and surveys,^61^ and then used in life‐cycle assessments (LCAs) or carbon accounting methodologies. These techniques are used to highlight hotspots of emissions and to focus attention on the most impactful activities. For example, using interviews, observations, and questionnaires, it was estimated that the 9 million fans in eight soccer tiers of England generated the equivalent of 2100 metric tons of CO_2_e (tCO_2_e) from waste sent to landfills during the 2012/13 season.^62^ This compares with 56,200 tCO_2_e from travel by spectators to/from games (i.e., more than 25 times greater than emissions from waste).^63^ However, these figures are trivial when compared with the CF of the whole activities of soccer sponsors in carbon‐intensive industries.^64^ Hence, some are calling for a more inclusive approach to CFs that extends beyond the fans, players, and sports clubs and their operations to reflect the climate impact of the whole ecosystem of stakeholders, including sponsors.^65^

### Emissions control/decarbonization

The carbon management hierarchy involves first avoiding carbon‐intensive activities, next reducing emissions, then replacing high‐ with lower‐carbon activities, after that managing carbon sinks to sequester emissions, and finally off‐setting. The last two steps in this chain are covered in the next section. Here, we provide examples of research into how sport can avoid, reduce, and replace carbon‐intensive actions.

The CF of the 2008 Beijing Olympic Games was estimated to be 0.77‒2.1 million tCO_2_e.^66^ This was accrued mainly from international/domestic flights, followed to a much lesser extent by the operation of venues, construction of new facilities, and local transport. Given the substantial CF of this event, other MSEs, and leagues (Table 1), it is unsurprising that much research concentrates on reducing the impact of travel by spectators and competitors.^67^ This can be achieved by switching modes of transport, increasing vehicle sharing, reducing the number and lengths of journeys taken, and improving driving and vehicle efficiency. The latter was integral to carbon emissions generated by Formula 1 Sunday races, which fell markedly during the period between 1992 and 1995 due to lighter and more efficient engines (although more “green” regulations in 2009–2011 had no discernible impact).^68^

Substantial cuts in CFs can be achieved by optimizing schedules to minimize the amount of travel between competition venues.^69^, ^70^ One analysis shows that using better logistics to shape the Formula 1 calendar could reduce carbon emissions by 44% when compared to the actual 2019 Grand Prix schedule.^71^ This covers all air, sea, and road transportation of equipment and tires (but excludes business travel, facilities, factories, and event operations). Improved logistics around food supply chains and distribution points for sport events can minimize carbon emissions too.^72^ Unfortunately, “smarter” schedules have not prevailed in soccer, with major tournaments now scheduled across whole continents. For example, rather than a single nation, 11 host cities spanning Dublin in the west and Baku in the east were involved in Euro 2020. It was also notable when two all‐English European Finals in 2019 were held in Madrid (Liverpool v Tottenham) and Baku (Arsenal v Chelsea).

Some have suggested that a transport optimization approach could even be taken globally when judging cities that have qualified to host a major event, such as the FIFA World Cup.^73^ The number of participating teams and the carbon intensity of tourist accommodation are important parts of the carbon calculation.^74^ The CF of fans and team travel may additionally be reduced by promoting low‐carbon travel options, greater vehicle‐sharing, and discouraging long‐distance air travel.^75^ Detailed analysis of transport modes and journeys before and during the Beijing Olympic Games showed greater use of mass transit and a 36% reduction in emissions per trip.^76^ More radically, the CF of travel to venues and on‐site consumption can be significantly reduced by holding events with no spectators in attendance.^77^ For instance, it is estimated that COVID‐19 restrictions on international spectators and cancellation of local ticket sales avoided 500,000 tCO_2_e at the Beijing 2022 Winter Olympics.^78^ Similarly, fewer officials, media, Olympic family, and marketing partners at the 2020 Tokyo Olympics^79^ reduced the CF by 129,686 tCO_2_e. Downsizing and rotating events among the same cities could also reduce emissions associated with new construction and visitors.^80^

Further emission reductions may be delivered through enhanced operational management and control systems of sports facilities to optimize heating, ventilation, air conditioning, lighting, appliances, and ICT systems.^81^ Stadiums, swimming pools, and sports centers—including those run by the educational sector^82^—attract attention given their high energy demands and scope for energy saving.^83^ Some studies develop methods for benchmarking building energy performance to identify comparative flaws in design or operation—for pools, the best baseline indicators of performance are visitor numbers and water area.^84^ Others use model simulations of pools^85^ and sports halls^86^ to evaluate energy efficiency gains from various scenarios of electromechanical and architectural retrofit (e.g., increased shading, insulation, and ventilation). Energy demands can be met by installing solar panels and/or wind turbines in stadiums.^87^

Waste management strategies may be evaluated to minimize emissions too—but sustainability practices vary greatly between teams.^88^ Auditing and modeling of waste scenarios for the University of Missouri football stadium in 2014 determined that elimination of edible food waste (especially beef) would reduce emissions by 103.1 tCO_2_e, and 100%‐recycling by 25.4 tCO_2_e.^89^ However, for a club to achieve carbon neutrality requires much more than waste management, a transport strategy, and improved energy efficiency (Table 2). The case of the Forest Green Rovers soccer team offers a template for others to follow.^90^ Their model of climate action includes a stadium powered by 100% renewable energy, electric vehicles and charging station, shirts made from bamboo waste and recycled plastic, and a vegan‐only menu. Reading F.C. is using shirts fabricated from recycled plastic bottles and features the “climate stripes” visualization of global warming.^91^

**TABLE 2:** Actions taken by the Forest Green Rovers soccer club to achieve carbon neutrality

Category	Action
Energy sources	Stadium powered by 100% renewable energy Use of solar panels
Energy efficiency	Automated lawn mowing by electric equipment
Transport	Electric charging station Team uses 100% electric vehicles Promotion of cycling, car sharing, and public transport for home and away supporters
Waste management	Shirts made from bamboo waste and recycled plastic Composting and recycling of used lawn mats Replacement of single‐use plastics
Food	Providing only vegan food for fans and players
Communications	Literal “greening” of official club colors Promoting actions to reduce the ecological footprint and rewarding well‐performing supporters Involving sponsors and business partners with green values plus organizing joint actions and promotions

*Source*: Adapted from Papp‐Vary and Farkas.^90^

Major sporting events, such as the Beijing 2008 Olympic Games, have been held up as an example of how to decarbonize a host city,^63^, ^92^ and even cited as a turning point for low carbon development (in China). The potential for low energy, low pollution, and low carbon development of the sport‐tourism sector has been widely discussed.^93^, ^94^, ^95^, ^96^ Advocates for “green sports” are calling for low‐carbon facilities and sports manufacturing, plus greater awareness and promotion of environmental concerns (including via sports celebrities).^97^ However, there are tradeoffs between the anticipated economic benefits from visitors to MSEs plus a longer‐term boost in tourist arrivals,^98^ versus the associated CFs from increased travel and resource consumption.^77^ Some are concerned that MSEs may divert scarce public and natural resources from other services^99^ and sustainability initiatives.^100^ Nonetheless, a few studies recognize that air pollution controls (such as traffic restrictions, reduced production at cement works, or even closure of polluting industries and construction sites) can have a cobenefit of smaller CFs. For example, steps taken to improve air quality during the Nanjing 2014 Youth Olympic Games yielded a 37% cut in carbon emissions.^38^

The Brisbane 2032 Olympics are striving to go a step beyond carbon neutrality to become the first climate‐positive Games.^101^ This will require reductions in greenhouse gases that exceed the direct and indirect CF of the event, plus other climate benefits for host communities. According to the hosts, the carbon budget will be aligned with and contribute to Queensland's emissions reduction and energy targets. These are for 50% renewable energy by 2030 and net zero emissions by 2050. Moreover, it is claimed that the sustainability ambitions of the Brisbane Games could catalyze a “more‐than‐human” approach to urban design and regeneration of the city.^102^

### Carbon sinks/offsetting

Sports fields, parks, and facilities may be designed and managed in ways that exploit their capacity to sequester carbon, thereby neutralizing emissions caused by their activities. For instance, detailed field measurements and laboratory tests show that the plant‐soil systems of golf courses can behave as carbon sinks. The amount of carbon accumulation depends on site‐specific factors, such as the choice of turfgrass species,^103^ the area of trees,^104^ relative mix of tees, rough, fairways, and greens,^50^ and age of the course.^105^ However, these sinks are countered by emissions from course vehicles and mowing, fertilizers and fungicides, green irrigation, decomposition of grass clipping, and waste disposal. The intensity of such management varies with the season causing carbon emissions to peak in summer. The largest source of emissions from a golf course in the UK was from vehicles, followed by fertilizers and fungicides, and irrigation.^106^ Considering all sources and sinks, the net carbon sequestration of this course was found to be between 0.455 tCO_2_e ha^−1^ year^−1^ (green) and 0.796 tCO_2_e ha^−1^ year^−1^ (mown rough). This compares with an average sink of 0.440 tCO_2_e ha^−1^ year^−1^ for modeled courses in Ohio.^107^ Depending on the management regime, a newly developed golf course could switch from a net carbon sink to a source within 30 years.

Carbon offsetting schemes should be a last resort when emissions are too costly or technically hard to reduce. The Delhi 2010 Commonwealth Games^108^ and Vancouver 2010 Winter Olympics^109^ were early promoters of voluntary carbon offsets (VCOs) for athletes, media, spectators, and sponsors traveling long distances to venues. Ideally, the income generated is used to purchase carbon credits for clean‐energy projects that create employment and serve local communities. For example, a solar water heater project was used to offset 246,200 tCO_2_e from activities at the Durban venue in the South Africa 2010 FIFA World Cup.^110^ Likewise, an unofficial Ohio State Athletics Carbon Offset Plan called for the installation of energy‐efficient products in the homes of low‐income communities.^111^ Alternatively, stadiums themselves can be used to generate renewable energy, saving substantial amounts of carbon and energy costs each year.^84^ The London 2012 Olympics initially pledged to offset *all* emissions generated by the event through investments in renewable energy projects in the developing world. These plans were later scaled back in favor of initiatives to promote behavior change and improved designs to eliminate emissions at source.^112^ Emissions associated with travel by competitors and spectators were offset by six projects run by BP Target Neutral.^113^

Some VCO schemes seek to capitalize on the assumed link between participants in outdoor sports, concern for the environment, and proenvironmental behavior. Some outdoor recreation companies now promote or provide VCO programs targeted at winter sports enthusiasts and trail runners. Research on the subject uses socioeconomic and household data to uncover predictors of VCO purchasing behavior. Evidence suggests that among runners, women, older participants, and households (rather than individuals) were most likely to buy offsets.^114^ Among participants of snow‐based activities in Canada, the most common characteristics were lower ages, higher education, a low carbon diet, inclination to outdoor pursuits, and awareness of VCO programs.^115^ More generally, the disconnect between VCO purchase and the time taken for tangible benefits to the climate represents key obstacles to their uptake.

**FIGURE 3:**
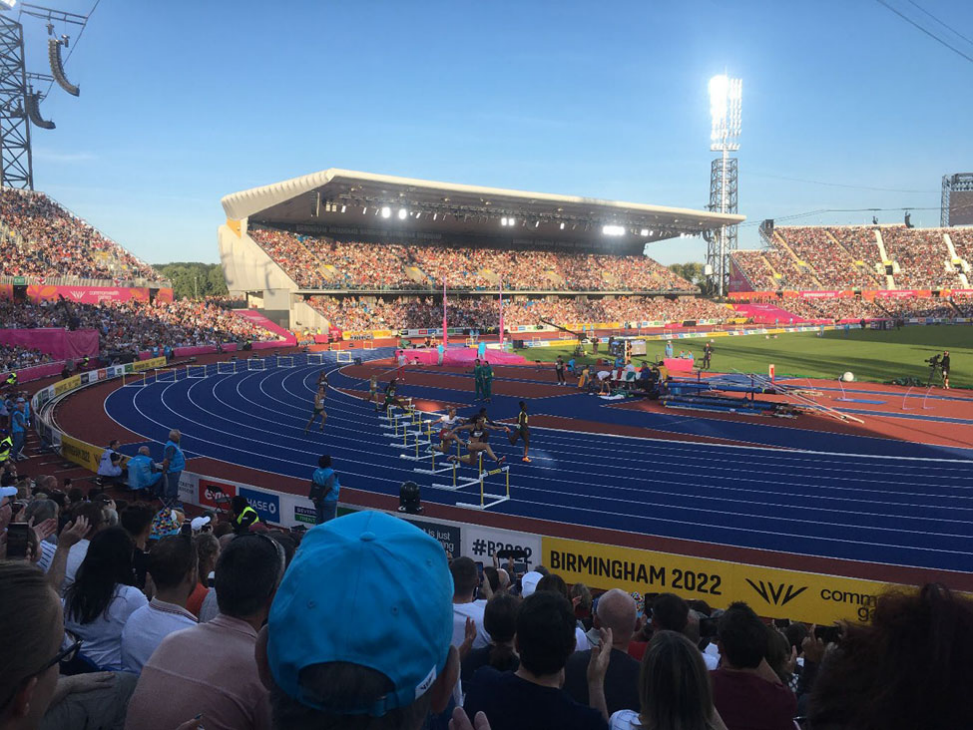
The Birmingham 2022 Commonwealth Games brought many benefits to the city but cost an estimated 240,000 tCO_2_e that will be offset over 35 years by new forest areas in the Midlands, UK. Photo: Author.

Local tree‐planting (“event greening”) has been widely adopted by hosts, such as the FIFA World Cup^107^ and Olympic Games,^116^ to counter CFs from new construction and activities. For example, the Birmingham 2022 Commonwealth Games (Figure 3) will offset emissions by planting 2022 acres of new forest within the Midlands, UK.^117^ More generally, the optimal choice of tree and shrub species should reflect local conditions and availability of irrigation water—with the possibility of using treated sewage water in arid regions.^118^ Tree‐planting may be a tangible mitigation action, but it can be challenging to measure the success of community forestry schemes or to guarantee the permanence of the sequestered carbon.^119^ This is because the carbon liability may take decades to neutralize. Standardization of carbon accounting techniques and measurement of above‐ground carbon stocks can help to keep track of the sink performance of urban green spaces.^113^, ^120^

### Behavior change

The final cluster of papers congregates around the theme of behavior change to mitigate the impact of sport on climate. This covers strategies for influencing transport decisions made by teams and participants, sustainable lifestyles, corporate social responsibility, and relationships with sponsors. Life‐cycle analysis of detailed data on the travel patterns of sport spectators and teams reveals the disproportionate climate impact of a minority of fans who travel by air.^72^ Strategies for reducing emissions from flights might include webcasts and big‐screen live sites at off‐site locations. Incentives that promote greater car occupancy rates for medium‐distance (<800 km) “away” journeys and cycling to local events would reduce the overall CF of supporters.^121^ This applies to those participating in mass participation (running) events.^122^ Influencing the travel mode choice of local spectators and competitors has less benefit because of their relatively small contributions to the overall CF of events. During the 2022 Major League Baseball season, the 30 teams will clock up more than a million miles of travel.^123^ Hence, rationalizing league boundaries and fixture schedules to reduce long‐haul travel by centralizing play‐offs, or clumping games between multiple teams in a single location, would have the largest impact on team CFs. Local reductions in emissions and operating costs can also be achieved through “Eco‐driver” training to reduce average speeds, idling time, and hard deceleration/acceleration of fleet vehicles.^124^

Community‐based organizations, such as schools, faith groups, and sports clubs, play important roles in promoting and nudging proenvironmental behaviors among their members.^125^, ^126^ Similarly, associative and mimetic behavior enable the diffusion of proenvironmental practices between teams and leagues.^127^ Empirical research provides evidence of the motivations behind behaviors and ways of strengthening the environmental impact of campaigns. For example, a study of Ipswich Town Football Club's “Save Your Energy for the Blues” campaign showed the importance of positive framing of actions and collective benefits.^128^ Fans were invited to pledge energy‐saving actions to offset ∼3000 tCO_2_e allowing the club to claim “first in the UK” carbon‐neutral status. Similarly, *Pledgeball* challenges soccer fans from opposing teams to save the most carbon emissions ahead of upcoming fixtures.^129^

Other research concentrates on the practical barriers to sustainability and or contradictions that might exist between awareness of environmental issues and behaviors. For example, despite awareness of environmental impacts, there are very low rates of recycling at soccer matches in Thailand because of insufficient attention to waste sorting.^130^ Elsewhere, the obstacle is sociocultural: in India, cycling is generally regarded as a lower‐class mode of transport (when compared with the car).^131^ Moreover, cycle rallies are framed more about health benefits and road safety than around climate action. For participants of winter sports who make long‐haul journeys, there is the irony of engaging in forms of transport that are harmful to the climate and hence to the snow on which their skiing depends.^132^ Surveys of personal responses to this tension reveal a willingness to pay for carbon offsets when folded into “green” ski passes^133^ or to car‐pool for repeat, short journeys to resorts.^134^ Although artificial snowmaking may be considered as an adaptation to climate change, it can reduce the attractiveness of a resort to summer skiers who are mindful of the associated high energy demand.^135^

Corporate branding and sponsorship of sport also shape consumer choices and are coming under closer scrutiny.^136^ One global survey itemized more than 250 prominent sports sponsorship deals with high‐carbon industries (e.g., fossil fuel companies, airlines, and car makers).^61^ This reflects concerns about the “greenwashing” of sport with contradictory signals to the climate action pledges made by clubs, governing bodies, and fans. Publicly visible sustainability league tables and reporting of air miles expose marked variations between clubs. According to research by BBC Sport,^137^ the top performing clubs in the English Premier League in February 2022 were Liverpool and Tottenham Hotspur (based on their policy and commitment, clean energy, energy efficiency, sustainable transport, single‐use plastic reduction or removal, waste management, water efficiency, plant‐based or low‐carbon food, biodiversity, education, communications, and engagement). Conversely, Aston Villa and Leeds United contributed the most emissions from preseason air travel in 2022.^138^

Conceptual frameworks are also emerging for self‐regulation and greater environmental sustainability of elite sport.^139^ While principles for ethical financing of sport are starting to take shape (Table 3), there are calls for greater engagement by researchers of ethics, philosophy, and ethnography.^133^ As legal actions proliferate against major emitters,^140^ some have imagined the possibility of indicting organizers of major sporting events for their reliance on high‐carbon sponsors.^141^

**TABLE 3:** Steps to be taken by clubs, governing bodies, and tournaments to phase out high carbon sponsors from sport

**Screen sponsors and owners** to exclude companies that promote high‐carbon lifestyles, products, and services, especially those in the automotive, airlines, and fossil fuel industries. **Sign the *UN Sport for Climate Action Framework*** and publish a detailed 10‐year plan to ensure that their own activities and that of their sport (including spectator travel) are decarbonizing. **Set annual emissions targets** with steps to achieve them and clear lines of responsibility for their delivery. **Establish robust monitoring** and reporting of progress against targets, reviewed by an independent body. **Exclude any global sports events, tours, or federations** that are not zero carbon by 2030. **Cut reliance on air travel** by holding fewer tournaments and by rationalizing the logistics of schedules and venues. **Make zero carbon plans a condition of public funding** of sporting organizations. **Prioritize support for low‐carbon, local, and grass‐roots sport** over the high‐carbon global and professional sport.

*Source*: Adapted from Tricarico and Simms.^64^

### Knowledge gaps and opportunities

Having navigated the research landscape for sport impacts on climate, this section considers some overarching issues before then highlighting key opportunities for further research in the concluding remarks.

First, it is important to acknowledge the limitations of our meta‐analysis. The search concentrated almost exclusively on materials published in the English language. This could contribute to some of the bias in the most researched sports and regions. Furthermore, “Sport,” “recreation,” “leisure,” and “tourism” are fuzzy and overlapping terms that can dictate whether or not a source is counted; likewise, there are interdisciplinary variations in the meaning and use of terms like “sustainable” and “green.” Even the distinction between sport equipment, sport apparel, and athleisure fashion was hard to discern in some cases. Our list of 25 most popular sports is open to question but at least it is declared explicitly and embraces the major organized and individual competitive sports globally.

Second, it is evident that this is a heterogeneous research area. On the one hand, MSEs, such as the Olympic Games and FIFA World Cup, attract a lot of research interest, as do popular sports, such as soccer, skiing, and golf (which is understandable given the bulk of surveyed publications are in the English language and the bias toward literature from Europe and North America). On the other hand, there is relatively little attention to global sports, such as cricket and volleyball, to studies in Africa, South America, and Australasia, or to qualitative research. The literature is also largely silent about the gendered impacts of sport on climate, and overall volumes of research output are modest even for the last decade. A few studies took advantage of COVID‐19 travel restrictions to have a fresh look at the benefits of avoided travel or to see the hiatus in sports attendance as an opportunity to reset thinking around league schedules.

Third, the evidence shows very mixed practices across the sports industry. There are some trail‐blazing teams, sports, and venues but much remains to be done by the sector from grass‐roots up to elite sports and their governing bodies. Exciting areas of research include the growing application of LCA to quantify emissions and prioritize mitigation efforts, imaginative ways of decarbonizing sports schedules through smarter logistics, and the power of clubs and athletes in affecting behavior change (especially around long‐haul travel). However, there needs to be more consistency in the treatment of Scope 3 emissions. For instance, should this include fan travel and at what point in the journey (particularly when combining attendance at a sport event with visiting family)? Increasingly, the nexuses between climate–sport–tourism–development or climate–sport–finance–ethics are recognized as legitimate areas for investigation and policy development.

Fourth, there is an emerging geopolitical dimension to debates around sport, climate change, and environmental degradation. This is because MSEs attract high‐level, international statespersons, providing opportunities for *ad hoc* diplomacy. To date, scholars have shown limited interest in the way diplomats leverage the incredible social and cultural reach of sport to shape global affairs as these pertain to climate change (as a form of soft power).^142^, ^143^, ^144^ These include: (1) use of MSEs for engaging in public diplomacy, whereby host nations/cities, individual statespersons, sporting organizations, and multinational as well as aspiring national businesses seek to improve their image/brand relative to climate action in the eyes of the watching world; (2) boycotting (or threatening to boycott) MSEs by nations, sponsors, sports teams, and individual athletes to make diplomatic points about climate change and related environmental crises; and (3) the use of MSEs by host nations, sporting bodies, nongovernmental organizations, and even individual athletes to draw attention to political causes, such as climate change. Under President Macron, the French Government has already announced plans to use the Paris 2024 Olympic and Paralympic Games to galvanize international commitments to reducing the impact of sport on the environment, including through actions to mitigate climate change and promote sustainability.^145^

Fifth, the literature does not adequately cover the tradeoffs between mitigation and adaptation, such as the use of air conditioning outdoors to manage extreme heat for the Qatar 2022 FIFA World Cup. There are also tensions between climate action and other planetary crises of biodiversity loss and pollution. Examples of this are producing fake snow with chemical additives that damage topsoil and plant growth; or switching to artificial turfs to avoid the challenges associated with drought, thereby interrupting patch proximity and pathways for insects and small animals.

Sixth, there is a danger of greenwashing in the realm of sport and climate mitigation. There are plenty of examples of high‐profile sustainability and carbon accounting reports *before* MSEs; follow‐up, longitudinal studies that ratify claims *after* events have ended are much harder to find. This is especially problematic when there is a high reliance on carbon offsets, such as new forests, which can take decades to counteract the emissions intensity of sport activity over much shorter periods. A few organizations, such as IOC, have provided organizing committees with methodological guidance, but there is a wider need for standardization of CF approaches within and between sports, and to undertake this work earlier in the planning/proposal cycle.

## CONCLUDING REMARKS

Based on the above observations and the body of evaluated literature, we close with several suggestions for further research. More studies are needed on the impacts of sport on climate from the perspective of:

1. Under‐represented continents (principally Africa and South America), global sports (badminton, cricket, rugby, tennis, and volleyball), and data‐gathering methods (qualitative).
2. Gendered aspects, especially given the lower resource implications of women's sports (due to smaller regional leagues, less budget for travel meaning, less impactful travel options, more local fanbases, and fewer international travelers attending events).
3. “Every‐day” and community‐level sport rather than elite sports, given the possibilities of scaling‐up and scope for behavior change.
4. Geopolitical influence of sport on climate mitigation (and adaptation) given the global reach and economic significance of MSEs, leagues, and clubs.
5. Affordable and easy‐to‐use LCA tools, standard guidance, and indicators that can be used by smaller clubs and individuals to measure their CFs and monitor progress in mitigating emissions.
6. Potential cobenefits and tradeoffs between mitigation‐adaptation efforts in sport, such as around logistics, choice of venues, sports equipment, and facilities.
7. Possible climate change litigation against the hosts and sponsors of MSEs, as well as energy‐intensive tournaments and global sports‐tourism providers.

Perhaps the time has come for a new Olympic motto. Rather than “*Citius, Altius, Fortius*” (“Faster, Higher, Stronger”), we now need “*Tardius, Proprius, Leviora*” (“Slower, Closer, Lighter”) footprints.

## AUTHOR CONTRIBUTIONS

R.L.W. conducted the analysis and led the writing of the paper. M.O. guided the methodology and conceptual development of the research. All authors contributed to the assessment of the literature and preparation of the manuscript.

## COMPETING INTERESTS

The authors have no relevant financial or nonfinancial interests to disclose.

### PEER REVIEW

The peer review history for this article is available at https://publons.com/publon/10.1111/nyas.14925.

## Supporting information

Supporting Information

## Data Availability

The dataset generated and analyzed during the current study is available from the corresponding author on reasonable request.

## References

[1] Research and Markets . (2022). Sports Global Market Report 2022, By Type, Revenue Source, Ownership. https://www.researchandmarkets.com/reports/5550013/sports‐global‐market‐report‐2022‐by‐type

[2] Sports Value . (2022). Coronavirus's economic impact on the Sports Industry. https://www.sportsvalue.com.br/en/coronaviruss‐economic‐impact‐on‐the‐sports‐industry/

[3] McBride, J. , & Manno, M. (2021). The economics of hosting the Olympic Games. https://www.cfr.org/backgrounder/economics‐hosting‐olympic‐games

[4] Killingstad, L. (2022). The most expensive World Cup in history. https://frontofficesports.com/the‐most‐expensive‐world‐cup‐in‐history/

[5] Foxman, S. , & Ismail, N. I. (2022). Football World Cup to add up to $17 billion to Qatari economy. https://www.bloomberg.com/news/articles/2022‐06‐22/soccer‐world‐cup‐to‐add‐up‐to‐17‐billion‐to‐qatari‐economy

[6] The King's Fund . (2022). The NHS budget and how it has changed. https://www.kingsfund.org.uk/projects/nhs‐in‐a‐nutshell/nhs‐budget

[7] Hulteen, R. M. , Smith, J. J. , Morgan, P. J. , Barnett, L. M. , Hallal, P. C. , Colyvas, K. , & Lubans, D. R. (2017). Global participation in sport and leisure‐time physical activities: A systematic review and meta‐analysis. Preventive Medicine, 95, 14–25. 27939265 10.1016/j.ypmed.2016.11.027

[8] Trendafilova, S. , McCullough, B. , Pfahl, M. , Nguyen, S. N. , Casper, J. , & Picariello, M. (2014). Environmental sustainability in sport: Current state and future trends. Global Journal on Advances Pure and Applied Sciences, 3, 9–14.

[9] McCullough, B. P. , Pfahl, M. E. , & Nguyen, S. N. (2016). The green waves of environmental sustainability in sport. Sport in Society, 19, 1040–1065.

[10] Schmidt, C. W. (2006). Putting the Earth in play: Environmental awareness and sport. Environmental Health Perspectives, 114(5), A286–A295. 16675410 10.1289/ehp.114-a286 PMC1459948

[11] Trendafilova, S. , & McCullough, B. P. (2018). Environmental sustainability scholarship and the efforts of the sport sector: A rapid review of literature. Cogent Social Sciences, 4, 1467256.

[12] Bernard, P. , Chevance, G. , Kingsbury, C. , Baillot, A. , Romain, A. J. , Molinier, V. , Gadais, T. , & Dancause, K. N. (2020). Climate change, physical activity and sport: A systematic review. Sports Medicine, 51, 1041–1059. 10.1007/s40279-021-01439-4 33689139

[13] McCullough, B. P. , Orr, M. , & Kellison, T. (2020). Sport ecology: Conceptualizing an emerging subdiscipline within sport management. Journal of Sport Management, 1, 1–12.

[14] Dingle, G. W. , & Stewart, B. (2018). Playing the climate game: Climate change impacts, resilience and adaptation in the climate‐dependent sport sector. Managing Sport and Leisure, 23, 293–314.

[15] Orr, M. , & Inoue, Y. (2019). Sport versus climate: Introducing the climate vulnerability of sport organizations framework. Sport Management Review, 22, 452–463.

[16] Dingle, G. , Dickson, G. , & Stewart, B. (2022). Major sport stadia, water resources and climate change: Impacts and adaptation. European Sport Management Quarterly. 10.1080/16184742.2022.2092169

[17] Orr, M. , & Schneider, I. (2018). Substitution interests among active‐sport tourists: The case of a cross‐country ski event. Journal of Sport & Tourism, 22, 315–332.

[18] Scott, D. , Steiger, R. , Rutty, M. , & Johnson, P. (2015). The future of the Olympic Winter Games in an era of climate change. Current Issues in Tourism, 18, 913–930.

[19] Scott, D. , Knowles, N. L. , Ma, S. , Rutty, M. , & Steiger, R. (2022). Climate change and the future of the Olympic Winter Games: Athlete and coach perspectives. Current Issues in Tourism. 10.1080/13683500.2021.2023480

[20] Steiger, R. , & Mayer, M. (2008). Snowmaking and climate change. Mountain Research and Development, 28, 292–298.

[21] DeChano‐Cook, L. M. , & Shelley, F. M. (2017). Climate change and the future of international events: A case of the Olympic and Paralympic Games. In B. P. McCullough & T. B. Kellison (Eds.), Routledge handbook of sport and the environment (pp. 66–78). Routledge.

[22] Miller‐Rushing, A. J. , Primack, R. B. , Phillips, N. , & Kaufmann, R. K. (2012). Effects of warming temperatures on winning times in the Boston Marathon. PLoS One, 7, e43579. 23049738 10.1371/journal.pone.0043579 PMC3458848

[23] Hodgson, J. R. , Elliott, S. , Chapman, L. , Hudson, C. , & Pope, F. D. (2020). The influence of meteorology and air quality on parkrun athletic performance. Parkrun Research. https://awrcparkrunresearch.wordpress.com/category/archived‐research‐projects/page/2/

[24] Brocherie, F. , Girard, O. , & Millet, G. P. (2015). Emerging environmental and weather challenges in outdoor sports. Climate, 3, 492–521.

[25] Townsend, M. , Mahoney, M. , Jones, J. A. , Ball, K. , Salmon, J. , & Finch, C. F. (2003). Too hot to trot? Exploring potential links between climate change, physical activity and health. Journal of Science and Medicine in Sport, 6, 260–265. 14609142 10.1016/s1440-2440(03)80019-1

[26] Matthews, T. , Perry, L. B. , Aryal, D. , Elmore, A. C. , Khadka, A. , Pelto, M. , Shrestha, D. , & Wilby, R. (2022). Weather on K2 during historic first winter ascent. Weather, 77, 49–52.

[27] Szymczak, R. K. , Marosz, M. , Grzywacz, T. , Sawicka, M. , & Naczyk, M. (2021). Death zone weather extremes mountaineers have experienced in successful ascents. Frontiers in Physiology, 12, 998. 10.3389/fphys.2021.696335 PMC8287323 34290622

[28] Orr, M. , Inoue, Y. , Seymour, R. , & Dingle, G. (2022). Impacts of climate change on organized sport: A scoping review. Wiley Interdisciplinary Reviews: Climate Change, 13, e760.

[29] Goldblatt, D. (2020). Playing against the clock. https://www.rapidtransition.org/resources/playing‐against‐the‐clock/

[30] Friedlingstein, P. , Jones, M. W. , O'sullivan, M. , Andrew, R. M. , Hauck, J. , Peters, G. P. , & Zaehle, S. (2019). Global carbon budget 2019. Earth System Science Data, 11, 1783–1838.

[31] United Nations Framework Convention on Climate Change . (2022). Sports for Climate Action. https://unfccc.int/climate‐action/sectoral‐engagement/sports‐for‐climate‐action

[32] United Nations Framework Convention on Climate Change . (2018). Sports for Climate Action Framework. https://unfccc.int/sites/default/files/resource/Sports_for_Climate_Action_Declaration_and_Framework.pdf

[33] Ferrari, R. (2015). Writing narrative style literature reviews. Medical Writing, 24, 230–235.

[34] Department for Business, Energy and Industrial Strategy . (2022). National Atmospheric Emissions Inventor. https://naei.beis.gov.uk/overview/ghg‐overview

[35] Sport Ecology Group . (2022). https://www.sportecology.org/research

[36] List 25 . (2019). Twenty‐five most popular sports in the world. https://list25.com/25‐most‐popular‐sports‐in‐the‐world/

[37] Logue, D. M. , Abiola, I. O. , Rains, D. , Bailey, N. W. , Zuk, M. , & Cade, W. H. (2010). Does signalling mitigate the cost of agonistic interactions? A test in a cricket that has lost its song. Proceedings of the Royal Society B: Biological Sciences, 277, 2571–2575. 10.1098/rspb.2010.0421 PMC2894931 20392727

[38] Graham, C. , Lynch, G. P. , English, T. , Hospers, L. , & Jay, O. (2021). Optimal break structures and cooling strategies to mitigate heat stress during a Rugby League match simulation. Journal of Science and Medicine in Sport, 24, 793–799. 34112612 10.1016/j.jsams.2021.04.013

[39] Hu, C. , Liu, C. , Hu, N. , Hong, J. , & Ai, X. (2022). Government environmental control measures on CO_2_ emission during the 2014 Youth Olympic Games in Nanjing: Perspectives from a top‐down approach. Journal of Environmental Sciences, 113, 165–178. 10.1016/j.jes.2021.04.016 34963526

[40] Miklautsch, P. , & Woschank, M. (2022). A framework of measures to mitigate greenhouse gas emissions in freight transport: Systematic literature review from a manufacturer's perspective. Journal of Cleaner Production, 366, 132883.

[41] Mishra, H. G. , Pandita, S. , Bhat, A. A. , Mishra, R. K. , & Sharma, S. (2022). Tourism and carbon emissions: A bibliometric review of the last three decades: 1990–2021. Tourism Review, 77, 636–658.

[42] Maibach, E. , Leiserowitz, A. , Cobb, S. , Shank, M. , Cobb, K. M. , & Gulledge, J. (2012). The legacy of climategate: Undermining or revitalizing climate science and policy? Wiley Interdisciplinary Reviews: Climate Change, 3, 289–295.

[43] Wilby, R. L. (2017). Why and how are carbon footprints measured? In: Climate change in practice. Cambridge University Press.

[44] International Olympic Committee . (2018). Carbon footprint methodology for the Olympic Games. https://library.olympics.com/Default/doc/SYRACUSE/184686

[45] Collins, A. , Flynn, A. , Munday, M. , & Roberts, A. (2007). Assessing the environmental consequences of major sporting events: The 2003/04 FA Cup Final. Urban Studies, 44, 457–476.

[46] Wicker, P. (2019). The carbon footprint of active sport participants. Sport Management Review, 22, 513–526.

[47] Pereira, R. P. T. , Filimonau, V. , & Ribeiro, G. M. (2019). Score a goal for climate: Assessing the carbon footprint of travel patterns of the English Premier League clubs. Journal of Cleaner Production, 227, 167–177.

[48] Loewen, C. , & Wicker, P. (2021). Travelling to Bundesliga matches: The carbon footprint of football fans. Journal of Sport & Tourism, 25, 253–272.

[49] Wu, Z. (2020). Haode evaluating the life‐cycle environmental impacts of polyester sports T‐shirts. In IOP Conference Series: Earth and Environmental Science (Vol., 474, No. 2, p. 022017). IOP Publishing.

[50] Hedayati, M. , Iyer‐Raniga, U. , & Crossin, E. (2014). A greenhouse gas assessment of a stadium in Australia. Building Research & Information, 42, 602–615.

[51] Chang, F. H. , Lin, T. C. , Huang, C. I. , Chao, H. R. , Chang, T. Y. , & Lu, C. S. (1999). Emission characteristics of VOCs from athletic tracks. Journal of Hazardous Materials, 70, 1–20. 10611425 10.1016/s0304-3894(99)00154-5

[52] Riches, D. , Porter, I. , Dingle, G. , Gendall, A. , & Grover, S. (2020). Soil greenhouse gas emissions from Australian sports fields. Science of the Total Environment, 707, 134420. 31863982 10.1016/j.scitotenv.2019.134420

[53] Tidåker, P. , Wesström, T. , & Kätterer, T. (2017). Energy use and greenhouse gas emissions from turf management of two Swedish golf courses. Urban Forestry & Urban Greening, 21, 80–87.

[54] Sampson, J. , Biesta, M. , Crapper, M. , Hall, I. , & Shepherd, A. (2013). Carbon dioxide accounting: 2014 Commonwealth Games Athletes' Village. Proceedings of the Institution of Civil Engineers‐Engineering Sustainability, 166, 150–160.

[55] Sahu, L. K. , Yadav, R. , & Pal, D. (2016). Source identification of VOCs at an urban site of western India: Effect of marathon events and anthropogenic emissions. Journal of Geophysical Research: Atmospheres, 121, 2416–2433.

[56] Koloszyc, H. (2016). A case study regarding the carbon footprint for one day trips to different ski destinations in the Jamtland region [Unpublished dissertation]. Mid Sweden University.

[57] Castaignède, L. , Veny, F. , Edwards, J. , & Billat, V. (2021). The carbon footprint of Marathon runners: Training and racing. International Journal of Environmental Research and Public Health, 18, 2769. 33803314 10.3390/ijerph18052769 PMC7967273

[58] Cooper, J. A. (2020). Making orange green? A critical carbon footprinting of Tennessee football gameday tourism. Journal of Sport & Tourism, 24, 31–51.

[59] Gillette, K. L. , Qian, Y. , Follett, R. F. , & Del Grosso, S. (2016). Nitrous oxide emissions from a golf course fairway and rough after application of different nitrogen fertilizers. Journal of Environmental Quality, 45, 1788–1795. 27695764 10.2134/jeq2016.02.0047

[60] Worden, H. M. , Cheng, Y. , Pfister, G. , Carmichael, G. R. , Zhang, Q. , Streets, D. G. , & Worden, J. R. (2012). Satellite‐based estimates of reduced CO and CO_2_ emissions due to traffic restrictions during the 2008 Beijing Olympics. Geophysical Research Letters, 39(14), L14802.

[61] Otto, I. , & Heath, E. T. (2009). The potential contribution of the 2010 Soccer World Cup to climate change: An exploratory study among tourism industry stakeholders in the Tshwane Metropole of South Africa. Journal of Sport & Tourism, 14, 169–191.

[62] Dosumu, A. , Colbeck, I. , & Bragg, R. (2014). Greenhouse gas emissions: Contributions made by football clubs in England. Atmospheric and Climate Sciences, 4, 642–652.

[63] Dosumu, A. , Colbeck, I. , & Bragg, R. (2017). Greenhouse gas emissions as a result of spectators travelling to football in England. Scientific Reports, 7, 6986. 28765597 10.1038/s41598-017-06141-y PMC5539281

[64] Tricarico, E. , & Simms, A. (2021). Sweat not oil: Why sports should drop advertising and sponsorship from high‐carbon polluters. https://www.rapidtransition.org/resources/sweat‐not‐oil‐why‐sports‐should‐drop‐advertising‐and‐sponsorship‐from‐high‐carbon‐polluters/

[65] Mabon, L. (2022). Football and climate change: A review of the scholarly evidence. 10.31235/osf.io/uz7r9

[66] Wu, D. , Zhang, S. , Xu, J. , & Zhu, T. (2011). The CO_2_ reduction effects and climate benefit of Beijing 2008 Summer Olympics green practice. Energy Procedia, 5, 280–296.

[67] Triantafyllidis, S. (2018). Carbon dioxide emissions research and sustainable transportation in the sports industry. Journal of Carbon Research, 4, 57.

[68] Mourao, P. R. (2018). Smoking gentlemen—How Formula one has controlled CO_2_ emissions. Sustainability, 10, 1841.

[69] Johnson, G. (2015). Striking out excess travel in major League Baseball: Reducing CO_2_ emissions due to the MLB schedule. https://nature.berkeley.edu/classes/es196/projects/2015final/JohnsonG_2015.pdf

[70] Wynes, S. (2021). COVID‐19 disruption demonstrates win‐win climate solutions for major league sports. Environmental Science & Technology, 55, 15609–15615. 34779201 10.1021/acs.est.1c03422

[71] Ross, H. (2020). Future sustainable practice for Formula 1 logistics [Unpublished Doctoral dissertation]. University of Strathclyde.

[72] Cai, Y. , Lin, Z. , Liu, L. , & Wang, Y. (2018). Demand forecast for Winter Olympic Games food cold chain logistics with carbon emissions consideration. In Proceedings of the 2018 10th International Conference on Information Management and Engineering (pp. 177–182).

[73] Pereira, R. P. T. , Camara, M. V. O. , Ribeiro, G. M. , & Filimonau, V. (2017). Applying the facility location problem model for selection of more climate benign mega sporting event hosts: A case of the FIFA World Cups. Journal of Cleaner Production, 159, 147–157.

[74] Pereira, R. P. T. , Filimonau, V. , & Ribeiro, G. M. (2020). Projecting the carbon footprint of tourist accommodation at the 2030 FIFA World Cup. Cleaner and Responsible Consumption, 1, 100004.

[75] Dolf, M. , & Teehan, P. (2015). Reducing the carbon footprint of spectator and team travel at the University of British Columbia's varsity sports events. Sport Management Review, 18, 244–255.

[76] Dong, S. , Shi, Q. , & Zhang, L. (2009). Greenhouse gas emissions reduction of commuting in Beijing Olympic Games. In 2009 12th International IEEE Conference on Intelligent Transportation Systems (pp. 1–6). IEEE.

[77] Mastromartino, B. , Ross, W. J. , Wear, H. , & Naraine, M. L. (2020). Thinking outside the ‘box’: A discussion of sports fans, teams, and the environment in the context of COVID‐19. Sport in Society, 23, 1707–1723.

[78] Mallapaty, S. (2022). China's Winter Olympics are carbon‐neutral—How? *Nature*, News, 4 February 2022. 10.1038/d41586-022-00321-1 35121813

[79] Ito, E. , Higham, J. , & Cheer, J. M. (2022). Carbon emission reduction and the Tokyo 2020 Olympics. Annals of Tourism Research Empirical Insights, 3, 100056.

[80] Müller, M. , Wolfe, S. D. , Gaffney, C. , Gogishvili, D. , Hug, M. , & Leick, A. (2021). An evaluation of the sustainability of the Olympic Games. Nature Sustainability, 4, 340–348.

[81] Kritzer, S. , Passegger, H. , Ayoub, T. , Liedtke, P. , Zellinger, M. , Stadler, M. , & Aghaie, H. (2021). Dekarbonisierung in Salzburgs Skigebieten–Entwicklung von Optimierungsalgorithmen und Energiemanagementsystemen zur Steigerung der Energieeffizienz, Minimierung von Emissionen und Optimierung von Flexibilitäten. e & i Elektrotechnik Und Informationstechnik, 138, 281–288.

[82] Ke, Y. (2021). Research on energy‐saving strategies of college stadiums and sports venues under the concept of low carbon development. In E3S Web of Conferences (Vol., 275, p. 02007). EDP Sciences.

[83] Elnour, M. , Fadli, F. , Himeur, Y. , Petri, I. , Rezgui, Y. , Meskin, N. , & Ahmad, A. M. (2022). Performance and energy optimization of building automation and management systems: Towards smart sustainable carbon‐neutral sports facilities. Renewable and Sustainable Energy Reviews, 162, 112401.

[84] Kampel, W. , Carlucci, S. , Aas, B. , & Bruland, A. (2016). A proposal of energy performance indicators for a reliable benchmark of swimming facilities. Energy and Buildings, 129, 186–198.

[85] Trianti‐Stourna, E. , Spyropoulou, K. , Theofylaktos, C. , Droutsa, K. , Balaras, C. A. , Santamouris, M. , Asimakopoulos, D. N. , Lazaropoulou, C. T. G. , & Papanikolaou, N. (1998). Energy conservation strategies for sports centers: Part B. Swimming pools. Energy and Buildings, 27, 123–135.

[86] Trianti‐Stourna, E. , Spyropoulou, K. , Theofylaktos, C. , Droutsa, K. , Balaras, C. A. , Santamouris, M. , Asimakopoulos, D. N. , Lazaropoulou, C. T. G. , & Papanikolaou, N. (1998). Energy conservation strategies for sports centers: Part A. Sports Halls. Energy and Buildings, 27, 109–122.

[87] Méndez, C. , & Bicer, Y. (2020). Towards a sustainable 2022 FIFA World Cup in Qatar: Evaluation of wind energy potential for three football stadiums. Energy Exploration & Exploitation, 38, 1893–1913.

[88] Francis, T. , Norris, J. , & Brinkmann, R. (2017). Sustainability initiatives in professional soccer. Soccer & Society, 18, 396–406.

[89] Costello, C. , McGarvey, R. G. , & Birisci, E. (2017). Achieving sustainability beyond zero waste: A case study from a college football stadium. Sustainability, 9, 1236.

[90] Papp‐Vary, A. F. , & Farkas, M. (2022). The world's first carbon neutral football club: The case study of Forest Green Rovers. Economic and Social Development: Book of Proceedings.

[91] Boudway, I. (2022). An English football club is adding global warming data to its kits. https://www.bloomberg.com/news/articles/2022‐08‐06/reading‐f‐c‐is‐adding‐global‐warming‐data‐to‐its‐uniform#:~:text=Greener%20Living‐,An%20English%20Soccer%20Club%20Is%20Adding%20Global%20Warming%20Data%20to,lapse%20image%20of%20rising%20temperatures

[92] Wu, J. , & Zhang, Y. (2008). Olympic Games promote the reduction in emissions of greenhouse gases in Beijing. Energy Policy, 36, 3422–3426.

[93] Bing, F. (2011). Study on development path of Shandong sports industry from the perspective of low‐carbon economy. Energy Procedia, 5, 879–883.

[94] Hu, D. , & Yang, Y. (2020). The development of marine sports tourism industry based on low‐carbon economy. Journal of Coastal Research, 112(SI), 97–99.

[95] Liu, P. H. , & Chen, X. (2014). Sustainable development of leisure sports tourism under the low‐carbon economy. Applied Mechanics and Materials, 675, 1781–1785.

[96] Yiqun, Y. , & Jian, L. (2010). Research on promotion of regional low‐carbon economy development by sports tourism industry of city circle. In Proceedings of the 7th International Conference on Innovation & Management (pp. 315–320).

[97] Chen, X. , Niu, J. , Nakagami, K. C. , Zhang, Q. , Qian, X. , & Nakajima, J. (2018). Green sports supporting a low‐carbon society: Inspiration from Japan. International Journal of Global Warming, 14, 61–80.

[98] Fourie, J. , & Santana‐Gallego, M. (2011). The impact of mega‐sport events on tourist arrivals. Tourism Management, 32, 1364–1370.

[99] Solberg, H. A. , & Preuss, H. (2007). Major sport events and long‐term tourism impacts. Journal of Sport Management, 21, 213–234.

[100] Giulianotti, R. , Darnell, S. , Collison, H. , & Howe, P. D. (2018). Sport for development and peace and the environment: The case for policy, practice, and research. Sustainability, 10, 2241.

[101] The State of Queensland Department of Environment and Science . (2021). Brisbane 2032 Climate Positive Games. https://www.des.qld.gov.au/climateaction/climate‐positive‐games

[102] Foth, M. , Kamols, N. , Turner, T. , Kovachevich, A. , & Hearn, G. (2022). Brisbane 2032: The promise of the first climate‐positive Olympics for regenerative cities. In R. Roggema (Ed.), Design for regenerative cities and landscapes: Rebalancing human impact and natural environment (pp. 227–248). Springer.

[103] Evers, M. , de Kroon, H. , Visser, E. , & de Caluwe, H. (2020). Carbon accumulation of cool season sports turfgrass species in distinctive soil layers. Agronomy Journal, 112, 3435–3449.

[104] Bartlett, M. D. , & James, I. T. (2011). A model of greenhouse gas emissions from the management of turf on two golf courses. Science of the Total Environment, 409, 1357–1367. 21288561 10.1016/j.scitotenv.2010.12.041

[105] Gautam, P. , Young, J. R. , Sapkota, M. , Longing, S. , & Weindorf, D. C. (2020). Soil carbon sequestration in bermudagrass golf course fairways in Lubbock, Texas. Agronomy Journal, 112, 148–157.

[106] Bartlett, M. D. , & James, I. T. (2011). Are golf courses a source or sink of atmospheric carbon dioxide? A modelling approach. Proceedings of the Institution of Mechanical Engineers, Part P: Journal of Sports Engineering and Technology, 225, 75–83.

[107] Selhorst, A. L. , & Lal, R. (2011). Carbon budgeting in golf course soils of Central Ohio. Urban Ecosystems, 14, 771–781.

[108] Sobhana, K. (2009). Games committee ropes in players to counter carbon footprint in 2010. https://indianexpress.com/article/cities/delhi/games‐committee‐ropes‐in‐players‐to‐counter‐carbon‐footprint‐in‐2010/

[109] Vancouver Organizing Committee for the 2010 Olympic and Paralympic Winter Games (VANOC) . (2010). Vancouver 2010 Sustainability Report 2009–10. https://stillmed.olympic.org/Documents/Reports/Official%20Past%20Games%20Reports/Winter/2010/ENG/Sustainability.pdf

[110] Diederichs, N. , & Roberts, D. (2016). Climate protection in mega‐event greening: The 2010 FIFA™ World Cup and COP17/CMP7 experiences in Durban, South Africa. Climate and Development, 8, 376–384.

[111] Clason, E. , Fricker, T. , Gadjigo, A. , Wendt, D. , & White, A. (2013). Ohio State's Athletic Carbon Offset Plan. OSU School of Environment and Natural Resources. https://core.ac.uk/download/pdf/159586271.pdf

[112] News and Analysis. (2011). London 2012 Olympics and Eurostar drop carbon‐offset schemes. Carbon management.

[113] BP . (2011). London 2012 spectators invited to set new carbon offset world record. https://www.bp.com/en/global/corporate/news‐and‐insights/press‐releases/london‐2012‐spectators‐invited‐to‐set‐new‐carbon‐offset‐world‐record.html

[114] Peterson, M. N. , Bondell, H. D. , Fratanduono, M. B. L. , Bigsby, K. , & McHale, M. (2013). Prediction indicators for voluntary carbon‐offset purchases among trail runners. Journal of Sport Behavior, 36, 264.

[115] Heintzman, P. (2021). The potential for voluntary carbon offset programs in the Canadian snow‐based outdoor recreation industry. Journal of Outdoor Recreation and Tourism, 36, 100422.

[116] Zhang, C. , & Li, Q. (2013). Apply MODIS products to analyze the changing carbon sink of Beijing before and after Olympics. Advanced Materials Research, 807, 1046–1051.

[117] Birmingham2022.com . (2022). Birmingham 2022 Commonwealth Games creating a carbon neutral legacy. https://www.birmingham2022.com/about‐us/our‐purpose/our‐legacy/sustainability/carbon‐neutral‐legacy

[118] Spanos, I. , Kucukvar, M. , Bell, T. C. , Elnimah, A. , Hamdan, H. , Al Meer, B. , Prakash, S. , Lundberg, O. , Kutty, A. A. , & AlKhereibi, A. H. (2022). How FIFA World Cup 2022 can meet the carbon neutral commitments and the United Nations 2030 Agenda for Sustainable Development?: Reflections from the tree nursery project in Qatar. Sustainable Development, 30, 203–226.

[119] Crabb, L. A. (2018). Debating the success of carbon‐offsetting projects at sports mega‐events. A case from the 2014 FIFA World Cup. Journal of Sustainable Forestry, 37, 178–196.

[120] Habib, S. , & Al‐Ghamdi, S. G. (2021). Estimation of above‐ground carbon‐stocks for urban greeneries in arid areas: Case study for Doha and FIFA World Cup Qatar 2022. Frontiers in Environmental Science, 9, 635365.

[121] Chard, C. , & Mallen, C. (2012). Examining the linkages between automobile use and carbon impacts of community‐based ice hockey. Sport Management Review, 15, 476–484.

[122] Triantafyllidis, S. , & Davakos, H. (2019). Growing cities and mass participant sport events: Traveling behaviors and carbon dioxide emissions. Journal of Carbon Research, 5, 49.

[123] Savant . (2022). 2022 MLB Travel Schedule. https://baseballsavant.mlb.com/visuals/map?team=&year=2022

[124] Rutty, M. , Matthews, L. , Scott, D. , & Matto, T. D. (2014). Using vehicle monitoring technology and eco‐driver training to reduce fuel use and emissions in tourism: A ski resort case study. Journal of Sustainable Tourism, 22, 787–800.

[125] Middlemiss, L. (2008). Influencing individual sustainability: A review of the evidence on the role of community‐based organisations. International Journal of Environment and Sustainable Development, 7, 78–93.

[126] Lehner, M. , Mont, O. , & Heiskanen, E. (2016). Nudging–A promising tool for sustainable consumption behaviour? Journal of Cleaner Production, 134, 166–177.

[127] Trendafilova, S. , Babiak, K. , & Heinze, K. (2013). Corporate social responsibility and environmental sustainability: Why professional sport is greening the playing field. Sport Management Review, 16, 298–313.

[128] Baldwin, R. (2010). Football and climate change: Strange bedfellows or a means of going beyond the usual suspects in encouraging pro‐environmental behavioural change? Local Environment, 15, 851–866.

[129] Amann, J. (2022). 1:0 for the environment: Engaging football fans on tackling climate change [Unpublished Masters Thesis]. University of Gothenburg.

[130] Atchariyasopon, K. (2017). Sustainable solid waste management in sports events: A case study of football matches in Thailand. Journal of Population and Social Studies, 25, 69–81.

[131] Joshi, R. , & Joseph, Y. (2015). Invisible cyclists and disappearing cycles: The challenges of cycling policies in Indian cities. Transfers, 5, 23–40.

[132] Stoddart, M. C. (2011). “If we wanted to be environmentally sustainable, we'd take the bus”: Skiing, mobility and the irony of climate change. Human Ecology Review, 18, 19–29.

[133] Haugom, E. , Malasevska, I. , Alnes, P. K. , & Mydland, Ø. (2021). Willingness to pay for “green skiing”. Journal of Hospitality and Tourism Management, 47, 252–255.

[134] Go‐Shred . (2022). Carsharing. https://go‐shred.com/carsharing/

[135] Demiroglu, O. C. , Dannevig, H. , & Aall, C. (2018). Climate change acknowledgement and responses of summer (glacier) ski visitors in Norway. Scandinavian Journal of Hospitality and Tourism, 18, 419–438.

[136] Mabon, L. (2021). Football financing, energy, and climate change. https://energyvalues.wordpress.com/2022/03/13/football‐financing‐energy‐and‐climate‐change/

[137] BBC Sport . (2022). How green are Premier League clubs and what are they doing to help? https://www.bbc.co.uk/sport/football/60196764

[138] BBC Sport . (2022). Should Premier League football clubs travel so far for pre‐season? https://www.bbc.co.uk/sport/football/62280394

[139] Gammelsæter, H. , & Loland, S. (2022). Code Red for Elite Sport. A critique of sustainability in elite sport and a tentative reform programme. European Sport Management Quarterly. 10.1080/16184742.2022.2096661

[140] Setzer, J. , & Vanhala, L. C. (2019). Climate change litigation: A review of research on courts and litigants in climate governance. Wiley Interdisciplinary Reviews: Climate Change, 10, e580.

[141] Renton, D. (2012). The trial of Lord Coe: As imagined by David Renton. Socialist Lawyer, 34–35.

[142] Murray, S. (2012). The two halves of sports‐diplomacy. Diplomacy & Statecraft, 23, 576–592.

[143] Murray, S. (2018). Sports diplomacy: Origins, theory and practice. Routledge.

[144] Dowse, S. (2018). Mega sports events as political tools: A case study of South Africa's hosting of the 2010 FIFA Football World Cup. In J. S. Rofe (Ed.) Sport and diplomacy: Games within games (pp. 70–86). Manchester University Press.

[145] Paris 2024 . (2019). Environmental ambition. https://www.paris2024.org/en/a‐pioneering‐ambition‐for‐the‐environment/

[146] Luthe, T. , Kägi, T. , & Reger, J. (2013). A systems approach to sustainable technical product design: combining life cycle assessment and virtual development in the case of skis. Journal of Industrial Ecology, 17, 605–617.

[147] Cheah, L. , Ciceri, N. D. , Olivetti, E. , Matsumura, S. , Forterre, D. , Roth, R. , & Kirchain, R. (2013). Manufacturing‐focused emissions reductions in footwear production. Journal of Cleaner Production, 44, 18–29.

[148] Formula 1 . (2019). F1 Sustainability Strategy. https://corp.formula1.com/wp-content/uploads/2019/11/Environmental-sustainability-Corp-website-vFINAL.pdf

[149] Chestney, N. (2012). London Olympics' emissions 28 percent lower than forecast: organizers. https://www.reuters.com/article/us-olympics-emissions-idUSBRE8BB0C320121212

[150] Rio 2016 Organising Committee for the Olympic and Paralympic Games . (2014). Carbon Footprint Management Report Rio 2016 Olympic and Paralympic Games. https://nachhaltigersport.files.wordpress.com/2016/04/carbon-footprint-management-report-rio-2016.pdf

[151] Collins, A. , Munday, M. , & Roberts, A. (2012). Environmental consequences of tourism consumption at major events: An analysis of the UK stages of the 2007 Tour de France. Journal of Travel Research, 51, 577–590.

[152] World Bank . (2022). CO_2_ emissions (metric tons per capita). https://data.worldbank.org/indicator/EN.ATM.CO2E.PC

