# Supplementary material for: The impacts of sport emissions on climate: Measurement, mitigation, and making a difference
Source: Ann N Y Acad Sci. 2022 Nov 15;1519(1):20–33. doi: 10.1111/nyas.14925 (PMC10098608; doi:10.1111/nyas.14925)
Supplement: Supplementary file 1 — Supporting Information [file NYAS-1519-20-s001.docx]

**Supporting Information**

**(A) Mitigation search terms**

("Carbon" OR "CO2" OR "Emission*" OR "Footprint*" OR "Mitig*" OR "Net Zero" OR "Greenhouse Gas*" OR “GHG” OR "Offset*" OR "Aerosol" OR "Decarb*")

**(B) Sport organizations and tournaments**

AND (“Sport*” or “Athlete*” or “coach” or “Olympic*” or “Paralympic*” or “Stadium” or “Stadia” or “FIFA World Cup” or “National Football League” or “NFL” or “National Basketball Association” or “NBA” or “Major League Baseball” or “MLB” or “Major League Soccer” or “MLS” or “National Women's Soccer League” or “NWSL” or “Women's National Basketball Association” or “WNBA” or “PGA” or “Ladies Professional Golf Association” or “LPGA” or “Tour de” or “Grand Slam” or “FIS” or “Association of Volleyball Professionals” or “AVP” or “World Surf” or “World Athletics” or “FINA” or “FIBA” or “Cricket World Cup” or “ICC World Cup” or “ICC T20 World Cup” or “Indian Premier League” or “Rugby Union” or “Rugby League” or “Australian Football League” or “English Premier League” or “La Liga" or “Bundesliga” or “Ligue 1” or “UEFA Champions League” or “Serie-A" or “J-League” or “A-League” or “FA Women's Super League” or “W-League” or “Nadeshiko League” or “Frauen-Bundesliga" or “Women's League” or “Rugby World Cup” or “Six Nations Rugby” or “Rugby Championship” or “Super Rugby” or “National Rugby League” or “Women's Rugby League” or “Japan Golf Tour” or “Sunshine Tour” or “Ladies European Tour” or “Ladies Asian Golf Tour” or “ALPG Tour” or “ATP World Tour” or “ITF Women's World Tennis Tour” or “National Collegiate Athletic Association” or “NCAA” or “Abbott Series” or “Super Bowl” or “race”)

**(C) Most popular sports^*,**, ***^**

AND (“Soccer” OR “Football” OR “Badminton” OR “Hockey” OR “Volleyball” OR “Basketball” OR “Tennis” OR “Cricket” OR “Table Tennis” OR “Baseball” OR “Golf” OR “Rugby” OR “Boxing” OR “Formula 1” OR “Motor Racing” OR “Cycling” OR “Lacrosse” OR “Athletics” OR “Track and Field” OR “Handball” OR “Bowling” OR “Martial Art*” OR “Ski” OR “Skiing” OR “Hurling” OR “Polo” or “Wrestling” OR “Parkrun*” OR “Marathon”)

* <https://www.realbuzz.com/articles-interests/sports-activities/article/top-10-most-popular-participation-sports-in-the-world/>

** <https://sportsbrief.com/facts/top-listicles/16715-revealed-top-15-popular-sports-world-2022/>

*** <https://list25.com/25-most-popular-sports-in-the-world/>
